# A Velocity Meter for Quantifying Advection Velocity Vectors in Large Water Bodies

**DOI:** 10.3390/s20247204

**Published:** 2020-12-16

**Authors:** Farzam Allafchi, Caterina Valeo, Angus Chu, Jianxun He, Waltfred Lee, Peter Oshkai, Norman Neumann

**Affiliations:** 1 Mechanical Engineering Department, University of Victoria, Victoria, BC V8W 2Y2, Canada; fallafchi@uvic.ca (F.A.); waltfredlee@uvic.ca (W.L.); poshkai@uvic.ca (P.O.); 2 Department of Civil Engineering, Schulich School of Engineering, University of Calgary, Calgary, AB T2N 1N4, Canada; achu@ucalgary.ca (A.C.); jianhe@ucalgary.ca (J.H.); 3 School of Public Health, University of Alberta, Edmonton, AB T6G 2J7, Canada; nfneuman@ualberta.ca

**Keywords:** velocity meter, stormwater pond, PIV, ADV, flow velocity, dye injection

## Abstract

A velocity meter was designed and built in order to meet market needs for an affordable instrument that measures the range of velocity magnitudes and direction experienced in medium- to large-sized water bodies. The velocity meter consists of a graduated plate with an injector protruding from the center and a camera held downward above the plate. Once the Dye Injection Velocity (DIV) meter is in the flow, dye is injected and the camera records the dye fluid transport. The recorded video is analyzed to determine the local flow velocity and direction. The DIV was calibrated for a range of velocities between 0.0094 m/s and 0.1566 m/s using particle image velocimetry (PIV) in a flow visualization flume. The accuracy of the instrument was found to be +6.3% and −9.8% of full scale. The coefficient of determination of the calibration curve was equal to 98%. Once calibrated, the DIV was deployed to the Inverness Stormwater pond in Calgary, Canada, for validation tests against an Acoustic Doppler Velocity (ADV) meter. During the validation tests, both flow velocity magnitude and direction were measured at several spatial points. The velocity magnitude results showed good agreement and the Mann-Whitney test showed no statistically significant difference (*p*-value > 0.05). At two spatial points, the differences between direction data were significant, which could be caused by the random errors involved in the validation test. However, the averaged data showed good agreement.

## 1. Introduction

Measuring streamflow velocity is an essential component in numerous applications and interests and several studies have been conducted to assess how flow velocity is measured in practice [1]. Labaky et al. (2009) introduced the Point Velocity Probe (PVP), which estimates flow velocity based on the travel time of a saline tracer between an injector and two detectors. The tracer is detected by measuring the conductivity of fluid (mainly water). The injector and detectors are located on the surface of a cylindrical probe. Experimental tests revealed that the instrument is capable of measuring flow velocities between 5 and 98 cm per day [2]. Another instrument for water flow velocity measurement is called the Horizontal Heat Pulse Flowmeter (HHPF), which consists of a probe containing a heater surrounded by a circular array of thermistors. A heat pulse is generated by the heater and diffuses radially. If the medium is stagnant, a symmetric distribution of heat is expected. However, the water displacement causes some advection of heated water, thus, leading to an asymmetric distribution of heat. Therefore in this situation, there is a difference between temperatures read by thermistors. By calibrating temperature differences versus water velocity, the velocity can be estimated [1]. Experimental results showed that HHPF can measure flow velocities between 3.5 × 10^−8^ to 3.5 × 10^−5^ m/s [3].

Colloidal borescope is another instrument for measuring flow velocity, consisting of magnifying lenses and miniature video cameras capable of observing natural particles in the flow. Using this method, both flow direction and magnitude can be measured. As particles pass beneath the lenses, a light source illuminates the particles. The video recorded by the camera is later analyzed to find the flow velocity. The best results with this instrument are obtained for particles moving with a rate below 12 × 10^−3^ m/s [1] and the total velocity range is below 25 × 10^−3^ m/s [4]. The velocity ranges of PVP, HHPF and colloidal borescope make them suitable for primarily groundwater applications and are not particularly suited for surface waters. 

However, there are other types of velocimetry methods that are more suitable for surface flow measurement. For example, propeller-based velocity meters, in which the flow causes rotation in a small propeller that is facing the flow direction head-on. The velocity range of such velocity meters is larger than PVP, HHPF and colloidal borescope. However, the range starts at a relatively higher value because the flow should be strong enough to overcome the friction in the mechanical parts of the instrument. The velocity range of a typical propeller-based velocity meter, used in research studies [5] is between 0.07 and 7 m/s [6]. This range makes the propeller-based velocity meters mostly suitable for measuring flow velocity of relatively fast flowing rivers but not other water bodies such as lakes and ponds because the low end of the range of the device might be higher than the flow velocity experienced in most parts of a large water body. 

On the other hand, Particle Image Velocimetry (PIV) is a global, quantitative method for monitoring fluid flow that yields a distribution of vectors over a plane or a volume [7,8]. In this method, tracer particles, so-called seeds, are illuminated with a known frequency. The light scattered by the tracer particles is recorded by a camera in a sequence of frames and the displacement of particles between light images is calculated through sophisticated post-processing [8]. This method has been used extensively in various research projects; however, PIV requires multiple instruments for which the setup is time consuming and complicated.

Moreover, acoustic Doppler velocimetry is a robust method for measuring flow velocity components in different directions. The working principles of Acoustic Doppler Velocity meter (ADV) consist of Doppler phase analysis of the high-frequency signal backscattered from suspended particles. Commercial ADVs have an emitter in the center that transmits short acoustic pulses at a fixed frequency. The disseminated acoustic pulse collides with suspended particles or bubbles, reflects back and is sensed by receivers that focus on a common region of the fluid called the ‘sampling volume.’ The physical size of the sampling volume for a commercial ADV is a cylinder less than one cm in both length and diameter [9]. The flow velocity is estimated analyzing the phase change between two successive coherent signals. ADVs are sophisticated instruments, however, they are easy to operate and are extensively used in different hydraulic field and laboratory studies [10]. The operating velocity range of a commercial ADV is between 0.001 and 4 m/s [9], which makes it useful for measuring flow velocities of rivers and other water bodies. However, they cost between 10K to 20K CAD, which makes it an unaffordable tool for many research and engineering projects. In addition, the footprint of ADVs—the physical size of the sampling volume—is very small compared to other velocimetry methods.

Recent research into stormwater ponds includes developing a model that can estimate bacteria level, such as *E. coli*, in stormwater in order to improve Best Management Practices in Canada [11]. In these types of studies, the majority of hydro-environmental modelling in the literature is not validated [12,13,14]. On the other hand, the importance of understanding the fluid flow field in such water bodies is highlighted [11]. Therefore, in attempts to validate modelling with observations of flow field velocity in the field, the Authors found no affordable velocity meter on the market that can be used to measure flow velocities in medium to large water bodies such as stormwater ponds. Therefore, a velocity meter was designed and built to address this need. The velocity meter or Dye Injection Velocity meter (DIV), is calibrated in a laboratory scale flume equipped with a PIV and further validated in a stormwater pond using an ADV. The method of dye injection was chosen for its simplicity, low cost and practical range of use. In terms of velocity range, the closest type of device to the DIV meter designed here is an ADV, however, the disadvantages associated with an ADV noted above make it less than ideal for the application of interest.

## 2. Materials and Methods

The DIV meter was designed and built in the Mechanical Engineering Machine Shop of the University of Victoria, Victoria, BC, Canada. The DIV has four main parts including an injector, a graduated plate, a camera and a structural frame holding the camera over the plate facing downward. The frame includes a pole and an arm with a clamp at the end to hold the camera. The pole is connected perpendicularly to the plate using a support beneath the plate. The operating principles involve injecting dye tracer into the flow and recording videos of the dye being transported by the flow. Electric red AmeriColor^®^ soft gel paste was diluted in water with the ratio of 2 to 1000 mL. The dye is injected from a vertical injector protruding from the center of a graduated plate. The plate is graduated with multiple circles, sharing the same center and diagonal lines that concur at the center. Each two consecutive circles and diagonal lines are 1 cm radially and 15 degrees angularly distanced, respectively. A 1000TVL Sony CCD endoscope camera, which was originally used as a pipe inspection camera, was held downward above the injector and plate with an arm. Several LED lights, already attached to the camera, illuminate the plate. Using a 30 mL syringe, the dye is injected and the camera records the movement in a 50 frame per second video. The video is saved and later analyzed frame by frame on a computer. It is assumed that the dye reaches flow velocity within 2 cm of the injection point. Therefore, the flow velocity was estimated by dividing 0.16 m length by the time it takes for the dye to travel from the second innermost circle to the outermost circle, which is 18 cm in radius.

The target range of velocity that the DIV can measure was set to be between 0.01 m/s and 0.2 m/s. At this range of velocity the flow over the plate stays laminar and the boundary layer thickness is determined by Equation (1) [15].

(1) δ=4.9LRe1/2,

where δ is the boundary layer thickness; L is the length of the plate and equal to 0.36 m; and Re is the Reynolds number. The maximum boundary layer thickness, which occurs at the end of the plate with a flow velocity equal to the lower bound of the velocity range, is equal to 0.028 m. Therefore, protruding the injector 3 cm higher than the plate is assurance that the viscous effects due to the plate on the transport of the dye is negligible.

### 2.1. Calibration Setup

**Figure 1:**
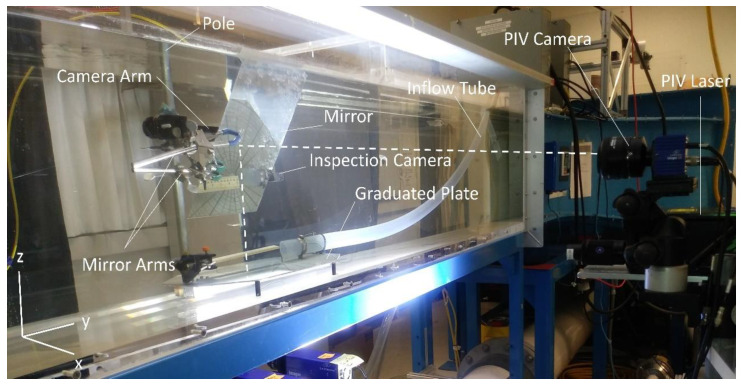
Calibration test setup at the Fluid Mechanics Laboratory, University of Victoria, Victoria, Canada.

The DIV meter was calibrated using a flow visualization flume equipped with a PIV system and located at the Fluid Mechanics Laboratory in the University of Victoria, Victoria, BC, Canada. The velocity operating range of the flume was not quite low enough for the test. Therefore, a 40 mm diameter flexible tube was placed near the injector and discharged the flow generated by a COPBB5 SHURflo Pedestal Mount centrifugal pump run by a Parker MPP1003D1E-KPSN motor. The tube was placed behind the injector so that the middle of the tube and the tip of the injector were at the same height. In this way, the injected dye was subjected to the highest discharged velocity. Various flow rates and thus flow velocities, were obtained by changing the rotor speed. The plane of interest for the PIV was parallel to the graduated plate, at a height equal to that of the center of the tube and the injector’s tip. Image shifting via a flat mirror was utilized for the PIV camera to overcome the optical restrictions. The mirror has an axial rotation around the *y*-axis at an angle of ϕ = 45° to prevent distortion. The camera field of view was set to capture the dye path in the middle to minimize any velocity shift [16]. Furthermore, the main velocity component, which is parallel to the rotation axis of the mirror (y-direction) is expected to be less sensitive to velocity shifts [17]. Moreover, the water level was set high enough to prevent any surface effects. Figure 1 demonstrates the calibration test setup configuration. The dashed line in the Figure 1 shows how the image was shifted by the mirror. The PIV camera was situated beside the flume recording the images and the laser was next to it at a lower height. It should be noted that the mirror and its supporting parts were only used for the calibration test and they are not part of the DIV.

The PIV system consists of Davis LaVision 10.0.1 software utilized for raw image acquisition and calculations of velocity vectors. Particles with a mean diameter of 10 µm were illuminated by the Quantel Evergreen Nd:YAG 532 nm wavelength dual pulsed laser. The laser beam was expanded into a planar sheet that illuminated the data acquisition plane (DAP). Images of the DAP were recorded using a LaVision XS 6M camera equipped with a lens with the focal length of 50 mm and a 532-nm narrow-band filter, at an acquisition rate of 13 Hz. The field of view of the images corresponded to the spatial domain was 160 mm × 80 mm. The resolution of raw image was 2752 pixels × 2200 pixels. The captured raw images were processed with a final interrogation window size of 64 × 64 pixels with 75% overlap applied throughout the multi-pass vector calculation process [18]. The spatial resolution of the resulting velocity vector field was 0.76 vectors/mm. Averaged velocity field was calculated by averaging 300 instantaneous velocity fields at each flow velocity. For the purpose of calibration, the PIV vectors from the averaged field were spatially averaged along a straight line between 2 and 18 cm far from the injector, respectively.

### 2.2. Validation Setup

**Figure 2:**
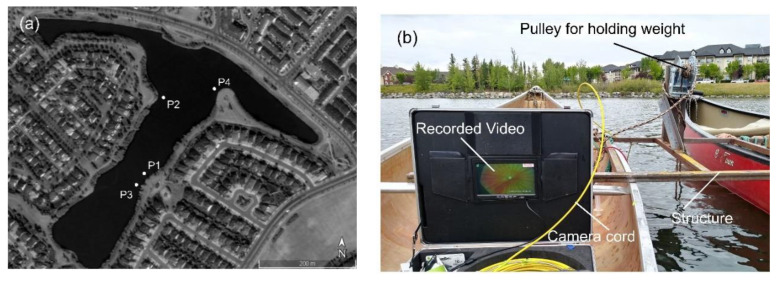
(**a**) Data collection locations in the Inverness pond (background photo extracted from Google Earth); (**b**) validation setup.

The DIV meter was deployed to the Inverness stormwater pond, Calgary, Canada, for validation tests. A structure was built to secure two canoes with a 1-m distance in between. The gap between the canoes was used to send the DIV down into the water. Four anchors were used to keep the boats in the data collection location. A heavy weight was sent to the bottom of the pond with a rope using a pulley attached to the structure. The rope was threaded through the pole of the DIV and tightened using the pulley. Figure 2 shows data collection locations and the validation tests’ setup. The velocity meter was kept in place using another rope attached to the camera arm. Velocity data from two locations on 27 August 2020 and four locations on 29 August 2020 were collected using both the DIV and FlowTracker2^®^, which is a commercial ADV [9]. The characteristics of the data collection locations are tabulated in Table 1. The ADV recorded two samples per second and the velocity was calculated based on 80 collected samples.

## 3. Results and Discussions

### 3.1. Calibration with the PIV

**Figure 3:**
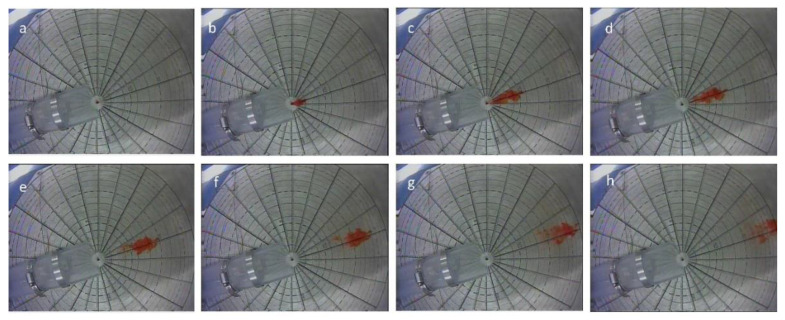
Transport of dye at an estimated speed equal to 0.14 m/s (prior to calibration) at (**a**) t = 0; (**b**) t = 0.380 s; (**c**) t = 0.619 s; (**d**) t = 0.710 s; (**e**) t = 0.928 s; (**f**) t = 1.244 s; (**g**) t = 1.520 s; and (**h**) t = 1.839 s.

**Figure 4:**
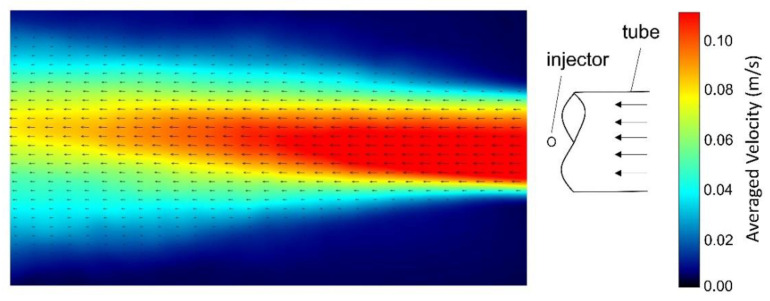
Particle image velocimetry (PIV) calculated velocity contour and vector field over the plate at a flow speed = 0.102 m/s.

In order to generate various fluid flows over the graduated plate for the calibration purpose, the rotor speed was altered to vary the flow. Following the rotor speed changes, enough time was given to let the flow reach steady state. Then, data were collected using both the DIV and PIV. Figure 3 illustrates a few sequences of a video recording the transport of dye in the flume. At each step, the dye was injected multiple times and the averaged elapsed time was considered for velocity calculations. The whole process, including changing rotor speed, multiple injections and PIV was repeated three times. Figure 4 contains an image of the velocity and vector fields resulting over the plate. The image is the time-averaged velocity vector field and the contours of time-averaged velocity magnitude in the horizontal plane located just above the plate (PIV plane of interest) obtained under repeatable laboratory conditions using PIV. The location of the injector point is indicated by a circle in the schematic in Figure 4. The data acquisition area extended from 2 cm downstream from the injector point (the right vertical boundary of the plot) and 18 cm downstream from the injector point (the left vertical boundary of the plot.) The velocity plot shows gradients of velocity across the top of the plate both in the streamwise and the transverse directions. These gradients can lead to errors in the case of very high flows, as discussed below.

**Figure 5:**
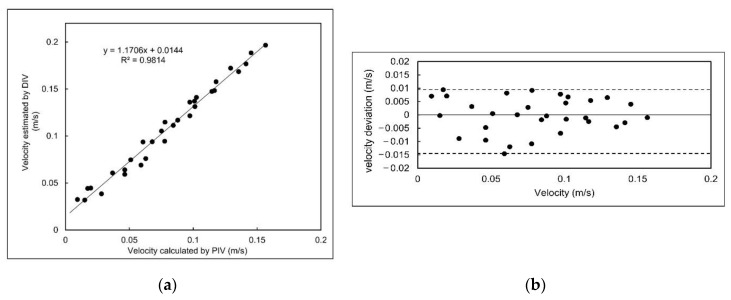
(**a**) Calibration curve; (**b**) plot of deviation data for Dye Injection Velocity (DIV) calibration.

Eleven data points in the second round and 10 data points during each of the other rounds were collected, making the total number of the data points equal to 31. The calibration curve of the DIV for all of the data was obtained and shown in Figure 5a. The R^2^ value of the calibration curve is more than 0.98, indicating a successful calibration and that the instrument can be reliably used to measure flow velocities.

**Figure 6:**
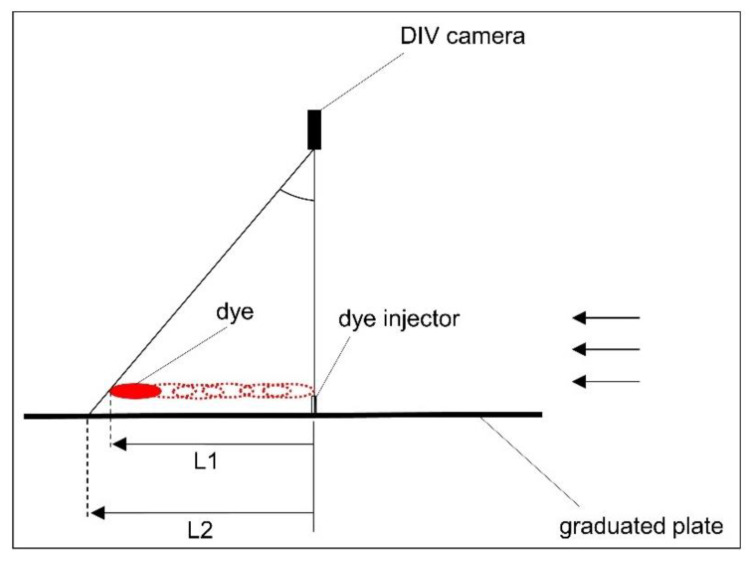
Actual distance travelled by the dye vs. the DIV camera estimation.

Comparing the velocity values estimated by the DIV and PIV, it was found that the DIV overestimated the velocity, for likely two reasons. First, due to turbulence and unsteady flow velocity in the dye pathline, the travel time that is recorded is the time for the fastest particles in the dye stream. Second, the dye is injected just above the plate and the DIV camera, which is located above the dye injector, observes the dye stream and plate beneath it from an angle that becomes more and more skewed as the dye progresses with the flow stream. This angle leads to a velocity overestimation and is illustrated in Figure 6. At the moment in time shown in Figure 6, DIV estimates the travelled distance to be equal to L2, whereas, the actual travel distance is L1 (and L1 < L2). This causes an underestimation in the dye travel time, which results in overestimation of flow velocity. 

The velocity target range had originally been set between 0.01 and 0.2 m/s and the low end of the target range was properly detected. However, no proper data was collected at flow velocities higher than 0.1566 m/s due to dye dispersion. This may have occurred because of the high velocity gradient present in the flume at high flow velocities, as shown in Figure 4. Hence, the DIV was calibrated for a velocity range between 0.0094 and 0.1566 m/s. Figure 5b shows the deviation of estimated velocity. The highest deviation from the calibration curve occurred at 0.0174 m/s and 0.0591 m/s, which were equal to +0.0094 m/s and −0.0145 m/s, respectively. Therefore, the accuracy of the instrument can be calculated as +6.3% and −9.8% of the output span. Higher uncertainties were observed near the low end of the detection range, with the highest equal to 28.7% corresponding to the low end of the range. As the flow velocity increased, the uncertainty decreased. For flow velocities higher than 0.06 m/s, 0.08 m/s and 0.1 m/s, the uncertainty was less than 15%, 10% and 5%, respectively.

The injection of the dye might induce some turbulence to the main flow that may contribute to a loading error. However, the calibration, through which the dye was injected multiple times, in separate, statistically independent tests, provides assurance that this effect is taken into account and its impact thereby reduced. In addition, the flume, in which the calibration tests were performed, was not able to generate very low flow velocities that fit the intended application. Therefore, low flows were generated using a rotor pump and tube added to the flume. However, the fluid flowed only locally and thus high velocity gradients existed at the periphery of the resulting jet flow. The turbulence due to the high velocity gradients is a source of calibration error and made it difficult to keep track of the dye and measure flow velocities higher than 0.1566 m/s. Therefore, it is believed that testing the DIV in a flume that is able to generate low velocity flows would not only result in a calibration with lower deviations but also might expand the calibration velocity range.

### 3.2. Uncertainty Analysis

There is some uncertainty associated with the recorded video of the DIV and how this uncertainty propagates to a final estimate of velocity can be calculated using error analysis. The distance that the dye traveled and the associated travel time were found by analyzing the video. The recorded video had a resolution of 720 × 288 pixels and 50 fps of frame rate. That corresponds to 0.5 mm and 0.02 s of distance and time uncertainty, respectively. These uncertainties propagate to the final estimate of velocity and this propagation is computed below. Velocity was calculated using Equation (2). The uncertainty in velocity due to the propagation of distance and time uncertainties can be calculated by Equation (3).

(2) $$V=\frac{d}{t}$$

(3) $$\sigma_{V}=\pm \sqrt{{\left(\left(\frac{\partial V}{\partial d}\right)\sigma_{d}\right)}^{2}+{\left(\left(\frac{\partial V}{\partial t}\right)\sigma_{t}\right)}^{2}},$$

where V, d and t are velocity, distance and time associated with the travel of the dye; and $\sigma_{V}$, $\sigma_{d}$ and $\sigma_{t}$ are the velocity, distance and time uncertainties, respectively. Equation (4) is the simplified form of Equation (3) with the appropriate substitution for the derivatives.

(4) $$\sigma_{V}=\pm \sqrt{{\left(\left(\frac{1}{t}\right)\sigma_{d}\right)}^{2}+{\left(\left(\frac{-d}{t^{2}}\right)\sigma_{t}\right)}^{2}},$$

The uncertainty of velocity due to the propagation of distance and time uncertainties was found to be between ± (6 × 10^−4^) m/s and ± (3.9 × 10^−3^) m/s. 

In addition, the velocity uncertainty can be analyzed with regard to the collected data. There are two kinds of uncertainty involved in the experiment, namely random and systematic uncertainties. The random uncertainty is calculated using Equation (5) [19]:

(5) $$P_{V}=\;\pm t\;\frac{S}{\sqrt{M}},$$

where P_V_ is the random uncertainty in velocity; t is the appropriate value of the t-distribution given the confidence level of the calculation; S is the standard deviation; and M is the sample size. The total uncertainty was calculated with Equation (6):

(6) $$W_{V}=\sqrt{B_{V}^{2}+P_{V}^{2}},$$

where W_V_ is the total uncertainty; and B_V_ is systematic uncertainty, which can be calculated from the accuracy of the instrument [19]. The data corresponding to the flow velocity of 0.0777 m/s after calibration were selected for uncertainty analysis because it has the largest sample size equal to 19. The random uncertainty for the mean value of the samples was calculated to be 0.0026 m/s with a 95% confidence level. The maximum systematic uncertainty was calculated by multiplying −9.8% by the full scale and found to be 0.0144 m/s. In general, the total uncertainty was calculated as 0.0146 m/s and this shows that the systematic uncertainty has a larger impact on the total uncertainty in the experiment as compared to the random uncertainty.

### 3.3. Validation with the ADV

**Figure 7:**
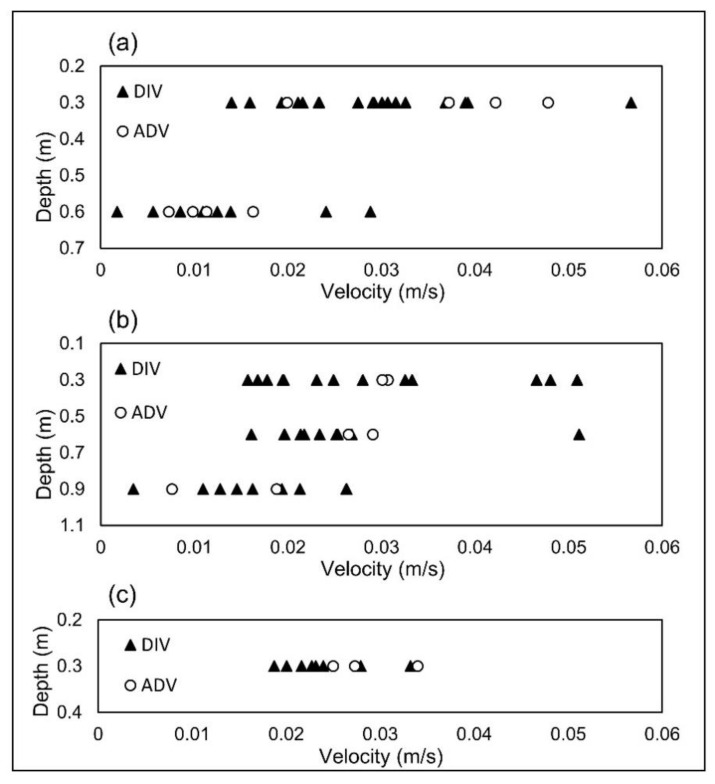
Validation tests results for flow velocity at (**a**) P1; (**b**) P2; and (**c**) P3.

Validation was performed in the Inverness stormwater pond and the results were compared with the results obtained from an ADV. The dye was injected multiple times and the velocity was averaged at each location. During the validation, flow direction was recorded as well as corresponding magnitude and the data were compared with that measured by the ADV. Figure 7 shows measured velocity magnitude results before averaging as estimated by both devices. Velocity data were collected from seven different spatial locations. However, the velocity magnitude at P4 was lower than the calibrated range and thus, the DIV was only used to find flow direction at this location.

**Figure 8:**
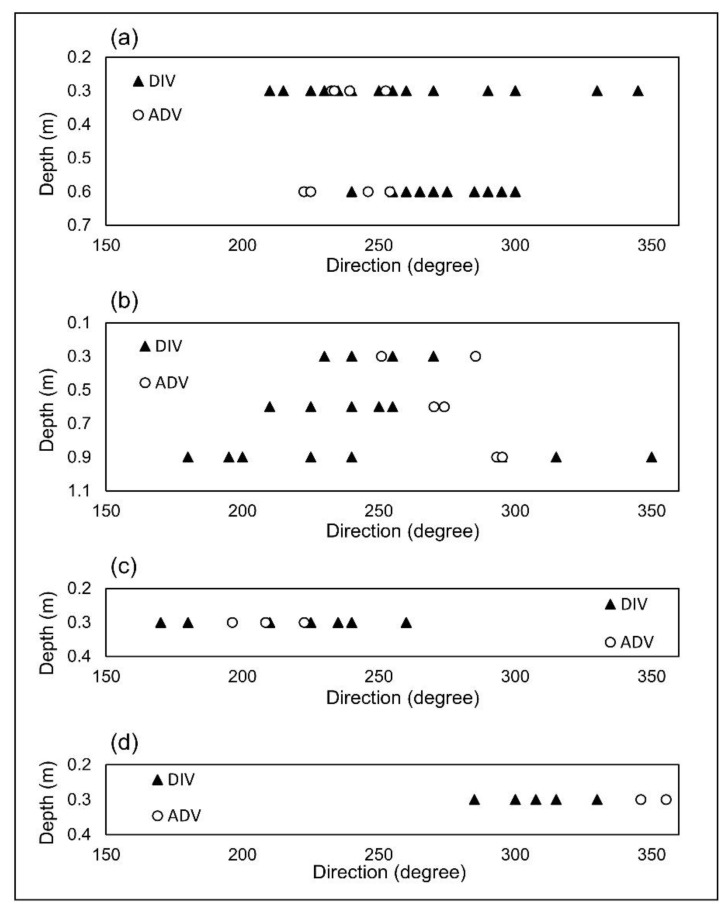
Validation tests results for flow direction at (**a**) P1; (**b**) P2; (**c**) P3; and (**d**) P4.

In order to compare the velocity magnitude data measured by the DIV and ADV, a Mann-Whitney test was performed. The *p*-value obtained for the difference in velocity magnitudes was larger than 0.05 in all of the spatial points; thus, indicating that the differences between the data are not statistically significant. In addition, the averaged values were compared and good agreement was observed. The lowest deviation between the averaged DIV and ADV occurred at the highest velocities recorded at P1 and P2 at both a 0.3 m depth. The percentage of those deviations are the lowest as well; equal to 4% and 5%, respectively. Conversely, the highest deviation between the averaged DIV and ADV measurements was equal to 0.0048 m/s, which occurred at location P3 at depth 0.3 m. However, the highest percentage in deviation was associated with P2 at a depth of 0.9 m and was equal to 27%. The velocity at this spatial point was close to the low end of the detection range and high uncertainty at the low end of the range was to be expected [19]. In addition, at this spatial point, the flow direction data were the most relatively dispersed as shown in Figure 8b. At low velocities, even small eddies can change velocity direction substantially. Therefore, low velocities may include higher random errors. 

There were other sources of systematic and random error involved in the validation tests. For example, during data collection, the data were not collected with the DIV and ADV simultaneously in order to avoid any interaction. However, specific attention was paid to shorten the time it took to switch from one device to another by as much as possible. While the flow in a medium to large water body like the Inverness Stormwater pond does not change instantly, it is possible that in the time it took to switch devices over the same collection point, the flow velocity may have changed, particularly in low velocity flows; thus adding to the random error. In addition, the boats were not perfectly still during data acquisition and they moved slightly with large waves or strong wind gusts. Furthermore, the boats might have had a loading impact on the flow by changing the velocity vector immediately around the boats. It should be noted that the structure held the DIV in the gap between the boats at a point equally distant from each boat in order to minimize the effects of the boats. 

The flow direction was shown in a trigonometric format (East = 0 and North = 90) in Figure 8. A Mann-Whitney test was also performed on flow directions estimated by the DIV and ADV. In 5 out of 7 spatial points the data were not significantly different (*p*-value > 0.05). However, at P1 and P2 and both at depth 0.6 m, the differences were significant. P1 at depth 0.6 m had a relatively low velocity, in which eddies can have a greater impact on the flow and thus change the flow direction. Other sources of error may have also contributed to difference in the data at P2 at a depth of 0.6 m. It should be noted that each ADV data point in Figure 7 and Figure 8 is the average velocity of 80 collected samples over 40 s with spikes filtered out automatically by the instrument. However, each data point corresponding to the DIV shows the flow velocity magnitude or direction for only one injection of the dye. For all of the spatial points the deviations of flow direction between the DIV and ADV measurements were less than 14%—the value equal to the corresponding deviation at P2 at a depth of 0.9 m. This demonstrates a good overall agreement in direction between the two devices. 

## 4. Conclusions

An efficient and easy to use Dye Injection Velocity meter was designed and built to measure fluid flow velocity magnitude and direction in medium to large water bodies at a relatively low cost. The DIV was calibrated for flow velocities between 0.0094 and 0.1566 m/s in a flume equipped with a PIV. An R^2^ value of 0.98 was obtained for the calibration curve and the accuracy of the instrument was calculated as between +6.3% and −9.8% of the output span. It was found that the uncertainty decreases with increasing velocity. Following the calibration tests, the DIV was validated against an ADV in a large stormwater pond and good agreement was achieved. Velocity magnitudes estimated by the DIV showed no statistically significant difference with those of ADV. The deviation between flow velocity measured by the DIV and that of ADV decreased with increasing velocity. During the validation tests, velocity direction was also measured. Although the difference between the flow direction measured by the DIV and ADV at two spatial points were significant, the deviation of the averaged values did not exceed 14% at any of the points. The results of the tests proved that the DIV can be used as a tool for velocity measurements in the range indicated here. However, further calibration tests in a flume that can generate very low velocities is recommended to provide better calibration accuracies.

## 5. Patents

The velocity meter presented in this work is filed at the United States Patent and Trademark Office as a provisional patent, US63/101,473.

## Figures and Tables

**Table 1:** Characteristics of data collection points.

Location	Depth from Water Level (m)	Date and Time
P1	0.3	27 August 2020 12:50 p.m.
P1	0.6	27 August 2020 01:35 p.m.
P2	0.3	29 August 2020 11:04 a.m.
P2	0.6	29 August 2020 11:25 a.m.
P2	0.9	29 August 2020 11:45 a.m.
P3	0.3	29 August 2020 02:08 p.m.
P4	0.3	29 August 2020 04:30 p.m.
